# Parental betaine supplementation promotes hepatic conversion of cholesterol to bile acids in offspring goslings with epigenetic modulation of CYP7A1 gene

**DOI:** 10.5713/ab.24.0857

**Published:** 2025-04-11

**Authors:** Shuai Ma, Liang Chen, Yan Wang, Lei Wu, Ruqian Zhao

**Affiliations:** 1 Key Laboratory of Animal Physiology and Biochemistry, College of Veterinary Medicine, Nanjing Agricultural University, Nanjing, China; 2 National Key Laboratory of Meat Quality Control and Cultured Meat Development, Nanjing, China

**Keywords:** Betaine, Cholesterol, CYP7A1, DNA Methylation, Goose, Liver

## Abstract

**Objective:**

Maternal betaine supplementation has been shown to affect offspring metabolism through epigenetic mechanisms in mammals. This study aimed to investigate whether parental betaine supplementation can exert intergenerational effects on hepatic cholesterol metabolism in offspring goslings, and the possible epigenetic modification associated with such effects.

**Methods:**

In this study, 450 female and 90 male Jiangnan White goose breeders, aged 39 weeks, were randomly assigned to three groups receiving basal (control, CON), or betaine-supplemented diets at low (LBT, 2.5 g/kg) or high (HBT, 5 g/kg) levels for 7 weeks. The breeder eggs laid in the last week were collected for incubation. Offspring goslings were slaughtered at 35 and 63 days of age to collect blood, bile, and liver samples for biochemical analysis and gene and protein expression studies.

**Results:**

The body weight of goslings in the LBT and HBT groups was significantly higher than that in the CON group (p<0.05). Serum and liver total cholesterol contents were significantly decreased in the HBT group (p<0.05), while the total bile acids contents in the liver and bile were significantly increased (p<0.05). These changes were associated with the upregulation of cholesterol-7 alpha-hydroxylase (CYP7A1) and bile salt export protein at both mRNA and protein levels (p<0.05). Additionally, parental betaine supplementation significantly decreased the DNA methylation level on the promoter region of the *CYP7A1* gene (p<0.05).

**Conclusion:**

These results indicate that parental betaine supplementation decreases hepatic cholesterol content in offspring goslings through epigenetic modulation of the CYP7A1 gene.

## INTRODUCTION

Cholesterol is a vital component of cell membranes and serves as a precursor for the biosynthesis of steroid hormones and bile acids, both of which are essential for health and disease [1,2]. The liver is the primary organ for cholesterol metabolism, acting as a regulatory hub to maintain cholesterol homeostasis. Unlike mammals, which can store excess cholesterol in adipose tissue [3], birds primarily regulate cholesterol homeostasis through hepatic clearance. In geese, serum cholesterol serves as a key precursor for bile acid synthesis, and steroid hormone production. Because birds lack functional brown adipose tissue for alternative lipid metabolism [4], they rely more on hepatic pathways for cholesterol conversion and excretion. This makes bile acid synthesis a crucial route for maintaining lipid balance and preventing cholesterol accumulation in geese. In chicken, cholesterol metabolism in muscle and brain is related to meat quality and aggressive behavior, respectively [5,6]. Disruption of cholesterol metabolism is a major factor contributing to fatty liver syndrome in chickens, leading to great economic losses in the poultry industry [7]. Therefore, regulating cholesterol metabolism could serve as a preventive strategy to benefit poultry health and the associated industry.

Hepatic cholesterol homeostasis is maintained through a dynamic balance between cholesterol biosynthesis and conversion into bile acids [1,8]. 3-Hydroxy-3-methylglutaryl-CoA reductase (HMGCR), a rate-limiting enzyme in cholesterol biosynthesis, is regulated by the key transcription factor sterol regulatory element-binding protein-2 (SREBP2) [1]. Additionally, cholesterol-7 alpha-hydroxylase (CYP7A1) and cholesterol-27 alpha-hydroxylase (CYP27A1) are critical rate-limiting enzymes responsible for converting hepatic cholesterol into bile acids [9]. These bile acids are subsequently transported from hepatocytes to the bile ducts via an ATP-dependent bile salt export pump (BSEP) and temporarily stored in the gallbladder [10]. Maternal nutrition interventions have been shown to affect cholesterol metabolism in offspring [11,12]. For instance, prenatal low-protein diet intake reduced the hepatic cholesterol content in weaning piglets by upregulating *CYP7A1* expression [13]. Similarly, maternal grape procyanidin exposure during the perinatal period elevated cholesterol content in offspring rats, which was associated with increased hepatic *HMGCR* expression [14]. However, the effects of parental betaine supplementation on hepatic cholesterol metabolism in offspring goslings remain to be elucidated.

Betaine, also known as trimethylglycine, is an effective methyl donor involved in various methylation processes within the body [15]. It is widely used as a feed additive in poultry diets to improve growth performance [16,17], alleviate heat stress [18,19], and regulate lipid metabolism [20]. Betaine is also critical for embryonic and fetal development, with numerous studies demonstrating its transgenerational effects on offspring performance in various species, including chickens [21], geese [22], pigs [23,24], and rodents [25,26]. Epigenetic mechanisms, such as DNA methylation, may link early-life nutrition to long-term alterations in metabolism and phenotype [27]. Betaine can activate DNA methylation via one-carbon metabolism, thereby modulating gene transcription [28]. Maternal betaine supplementation has been shown to enhance hepatic cholesterol accumulation in offspring rats, accompanied by hypermethylation of the *CYP7A1* gene promoter [29]. Additionally, maternal betaine exposure induced hypermethylation of differentially methylated regions on the *IGF-2* gene, resulting in elevated *IGF-2* expression in the hippocampus of neonatal piglets [30]. However, whether betaine supplementation in goose breeders affects the hepatic cholesterol metabolism of offspring goslings through DNA methylation remains unknown.

This study aims to investigate whether parental dietary betaine supplementation can affect hepatic cholesterol metabolism in offspring goslings, and how such effects, if any, are related to DNA methylation on the promoter of related genes.

## MATERIALS AND METHODS

### Ethics statement

All the experiments were approved by the Animal Ethics Committee of Nanjing Agricultural University. The project number is 31972638. The sampling procedures followed the “Guidelines on Ethical Treatment of Experimental Animals” (2006) No. 398 set by the Ministry of Science and Technology, China.

### Experimental design and diets

A total of 540 Jiangnan White goose breeders (450 females and 90 males at 39 weeks of age) were raised in the Geese breeding farm of Jiangsu Lihua Animal Husbandry Co., Ltd. Geese were randomly allocated into three groups, with five replicates each. Geese of each were raised in separate floor pens (4 m × 4 m) with 30 females and 6 males per pen mixed to allow natural mating. The control group (CON) received a basal diet, and the other groups received betaine-supplemented diets at low (LBT, 2.5 g/kg) or high (HBT, 5 g/kg) levels for 7 wks. Betaine was purchased from Beijing Xin Dayang Co., Ltd., 75% purity. The management of goose breeders was carried out under the company guidelines. The temperature of the room was maintained at 26°C, and the light regime of 16L:8D. Geese was allowed free access to feed and water throughout the study. The ingredient composition and nutrient contents of the diets are shown in Table 1.

Fertilized eggs laid last week were obtained from each group and incubated at a commercial hatchery. One-day-old goslings from each group were manually sexed and distributed into brood cages. The goslings were raised until 21 d and transferred to separate floor pens (4 m × 3 m) from 22 to 63 d. Each group contained 5 replicates with 32 goslings per pen (half male and half female). All goslings had ad libitum access to water and diet (Table 1) in an environmentally controlled room with continuous lighting. The temperature of the room was maintained at 32°C for the first week and gradually decreased by 1°C every 2 d until 21 to 22°C, with the humidity kept at 30% to 50%.

At 35 and 63 d, two goslings were randomly selected from each replicate and sacrificed by rapid decapitation according to the American Veterinary Medical Association Guidelines for Animal Euthanasia: 2013 edition. Blood samples were collected from the wing vein using a sterile syringe. Samples, including blood, bile, and liver, were stored at −80°C for further analysis.

### Biochemical parameters examination

Concentrations of total cholesterol (TCHO) and total bile acids (TBA) in the liver were extracted using the methods described previously [31,32]. The extracts together with bile and serum samples were used for determining the TCHO (H202) and TBA (H101T) as well as alanine transaminase (ALT, H001), aspartate aminotransferase (AST, H002) and lactate dehydrogenase (LDH, H008) concentrations with an automatic biochemical analyzer (Hitachi 7020, Hitachi, Tokyo, Japan). The above commercial assay kits were purchased from Meinkang Biotech Co., Ltd. (Ningbo, China).

### RNA isolation and real-time polymerase chain reaction for mRNA quantification

About 40 mg of liver samples were extracted using 1 mL TRIzol reagent (TSP401; Tsingke Biotech Co., Ltd, Nanjing, China) to obtain high-quality RNA, and 1 μg of RNA was reverse transcribed to cDNA (RK20429; ABclonal Technology Co., Wuhan, China). Diluted cDNA (1:20, vol/vol) was used for real-time polymerase chain reaction (PCR) with PerfectStart Green qPCR SuperMix (AQ601-02; TransGen Biotech Co., Ltd., Nanjing, China), which was performed with Quant Studio 6 Flex Real-Time PCR System (Applied Biosystems, Foster City, CA, USA). Peptidylprolyl isomerase A was used as an internal reference to calibrate and normalize the data. Data were analyzed using the method of 2^−ΔΔCT^. All primers (Table 2) were synthesized by Tsingke Biotechnology (Beijing, China).

### Total protein extraction and western blotting

Total protein was extracted from 50 mg of frozen liver samples and homogenized with RIPA buffer containing the protease inhibitor cocktail (B14001, Selleckchem). The homogenates were centrifuged at 12,000 rpm for 20 mins at 4°C. Protein concentration was determined using the BCA protein assay kit (23225, TransGen, Beijing, China). Forty micrograms of protein were loaded on 6% to 10% sodium dodecyl sulfate polyacrylamide gel electrophoresis gels for electrophoresis. Western blot analysis for farnesoid X receptor (FXR) (A24015, ABclonal, diluted 1:1,000), CYP7A1 (AB79847, Abcam, diluted 1:1,000), CYP27A1 (BS2192, Bioworld, diluted 1:1,000), BSEP (A8467, ABclonal, diluted 1:1,000), HMGCR (BS6625, Bioworld, diluted 1:1000), SREBP2 (14508-1-AP, Proteintech, diluted 1:1000), LXRα (A2141, ABclonal, diluted 1:1000). β-actin (AC026, ABclonal, diluted 1:100,000) was used as internal control. Images were captured by VersaDoc 4,000 MP system (Bio-Rad, Hercules, CA, USA) and the band density was analyzed by Image J software.

### Methylated DNA immunoprecipitation analysis

Methylated DNA immunoprecipitation (MeDIP) analysis was carried out as previously described [21]. Briefly, purified genomic DNA was extracted from liver samples and sonicated to produce an average size of 300 base pairs. One microgram of fragmented DNA was heat-denatured and immunoprecipitated with an anti-5 mC antibody (ab10805, Abcam, diluted 1:1,000). A portion of the denatured DNA was preserved as input DNA. The immune complexes were captured by pretreated protein G agarose beads (sc-2003; Santa Cruz Biotechnology, Inc., San Diego, CA, USA). Finally, the immunoprecipitated DNA was purified and used as a template to amplify the promoter sequences of *CYP7A1* and *BSEP* genes with specific primers (Table 2). Data were normalized against the input and presented as the fold change relative to the average value of the CON group.

### Statistical analysis

Data were analyzed by one-way ANOVA using IBM SPSS 20.0 software (SPSS Inc., Chicago, IL, USA). The differences among groups were examined by the Tukey test, which was considered statistically significant when p<0.05. For MeDIP data analysis, Student’s t-test was used to examine the difference between the CON and HBT groups. Data are presented as means±standard error of the mean.

## RESULTS

### Body weight, liver weight, and biochemical parameters in serum, bile, and liver

**Figure 1:**
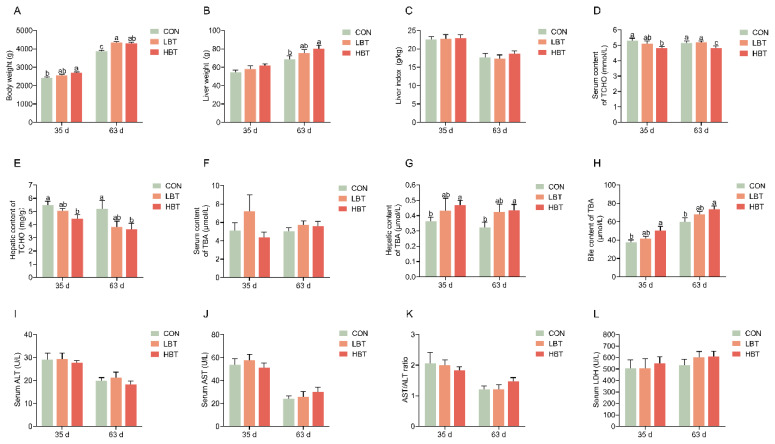
Effects of parental betaine supplementation on body and liver weights and biochemical parameters in serum, bile, and liver in offspring goslings with different ages. (A) Body weight. (B) Liver weight. (C) Liver index. (D) Serum content of TCHO. (E) Hepatic content of TCHO. (F) Serum content of TBA. (G) Hepatic content of TBA. (H) Bile content of TBA. (I) Serum content of ALT. (J) Serum content of AST. (K) AST/ALT ratio. (L) Serum content of LDH. Values are presented as mean±SEM (n = 10). Bars marked with different superscripts are significantly different (p<0.05). LBT, betaine-supplemented diets at low; HBT, betaine-supplemented diets at high; TCHO, total cholesterol; TBA, total bile acids; ALT, alanine transaminase; AST, aspartate aminotransferase; LDH, lactate dehydrogenase; SEM, standard error of the mean.

Compared to the CON group, the body weight in the HBT group showed a significant increase at 35 d (Figure 1A) (p<0.05), while both LBT and HBT groups had greater body weight at 63 d (Figure 1A) (p<0.05). The liver weight in the HBT group was significantly increased at 63 d (Figure 1B) (p<0.05), whereas there was no significant difference in liver index (Figure 1C). The concentrations of TCHO in serum and liver were significantly reduced in the HBT group at both 35 and 63 d (Figures 1D, 1E) (p<0.05). There were no significant differences among the three groups in the serum concentrations of TBA (Figure 1F). On the contrary, compared to the CON group, the HBT group showed a significant increase in TBA concentrations in the liver and bile at both 35 and 63 d (Figures 1G, 1H) (p<0.05). In addition, Parental betaine supplementation did not significantly change the serum parameters of ALT, AST, and LDH (Figures 1I–1L).

### Expression of hepatic cholesterol metabolism related-genes

**Figure 2:**
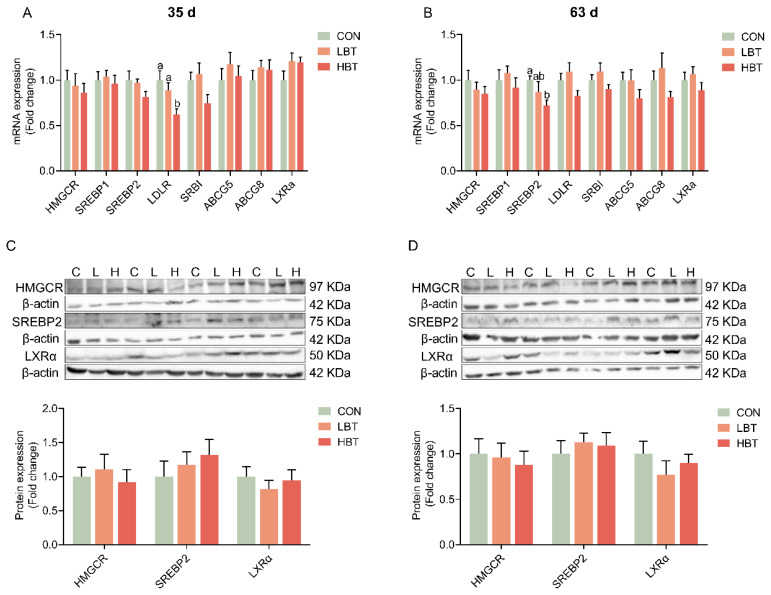
Effects of parental betaine supplementation on hepatic expression of cholesterol metabolic genes in offspring goslings with different ages. (A) Hepatic mRNA expression of genes involved in cholesterol metabolism at 35 d (n = 10). (B) Hepatic mRNA expression of genes involved in cholesterol metabolism at 63 d (n = 10). (C) Protein expression of cholesterol metabolism genes at 35 d (n = 8). (D) Protein expression of cholesterol metabolism genes at 63 d (n = 8). Values are presented as mean±SEM. Bars marked with different superscripts are significantly different (p<0.05). LBT, betaine-supplemented diets at low; HBT, betaine-supplemented diets at high; SEM, standard error of the mean.

At 35 d, low density lipoprotein receptor (LDLR) mRNA expression was significantly decreased in the liver of the HBT group (Figure 2A) (p<0.05). At 63 d, the SREBP2 mRNA expression was significantly decreased in the HBT group (Figure 2B) (p<0.05). Other genes did not show a significant difference (Figures 2A, 2B). Similarly, parental betaine supplementation also did not affect the protein expression of HMGCR, SREBP2, and LXRα in the liver (Figures 2C, 2D).

### Expression of hepatic bile acids metabolism related-genes

**Figure 3:**
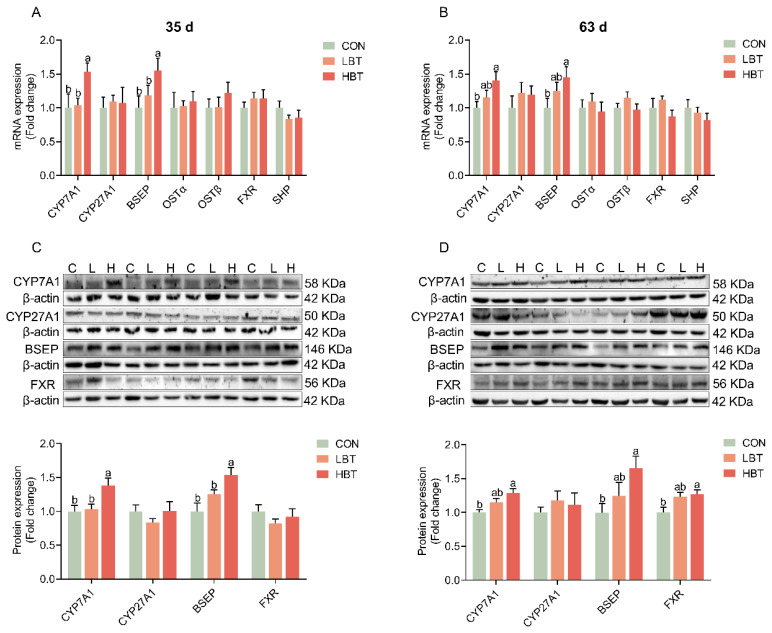
Effects of parental betaine supplementation on hepatic expression of bile acid synthesis and transport-related genes in offspring goslings with different ages. (A) Hepatic mRNA expression of genes involved in bile acid metabolism at 35 d (n = 10). (B) Hepatic mRNA expression of genes involved in bile acid metabolism at 63 d (n = 10). (C) Protein expression of bile acid metabolism genes at 35 d (n = 8). (D) Protein expression of bile acid metabolism genes at 63 d (n = 8). Values are presented as mean±SEM. Bars marked with different superscripts are significant differences (p<0.05). LBT, betaine-supplemented diets at low; HBT, betaine-supplemented diets at high; SHP, small heterodimer partner; BSEP, bile salt export pump; FXR, farnesoid X receptor; SEM, standard error of the mean.

At 35 and 63 d, the CYP7A1 and BSEP were significantly increased at both mRNA and protein levels in the liver of the HBT group (Figures 3A–3D) (p<0.05). Concurrently, hepatic protein expression of bile acids receptor FXR was also significantly upregulated in the HBT group at 63 d (Figure 3D) (p< 0.05).

### MeDIP analysis for DNA methylation status on the promoter of affected genes

**Figure 4:**
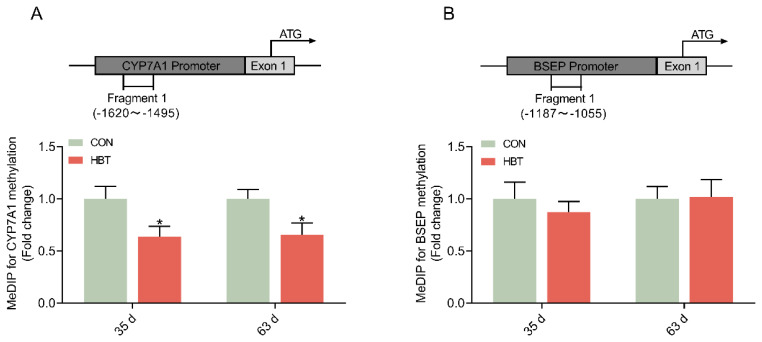
Effects of parental betaine supplementation on DNA methylation status at the promoter of affected genes with different ages. (A) DNA methylation on the promoter of the CYP7A1 gene. (B) DNA methylation on the promoter of the BSEP gene. Values are presented as mean±SEM (n = 6). * p<0.05. SEM, standard error of the mean.

We previously reported that parental betaine supplementation could enhance hepatic methionine metabolism and methyl transfer gene expression in the offspring goslings [22]. Next, the MeDIP-PCR method was used to analyze the genomic DNA isolated from the liver revealing that the DNA methylation status of the promoter region for *CYP7A1* gene was hypomethylated in the HBT group at both 35 and 63 d (Figure 4A) (p<0.05). No significant alterations were observed in the promoter region of the *BSEP* gene (Figure 4B). Because no CpG islands were found within the 5’ flanking sequence of the *HMGCR*, *SREBP2*, *LDLR*, and *FXR* genes in the goose, therefore we excluded these genes from methylated DNA immunoprecipitation.

## DISCUSSION

In the present study, parental betaine supplementation exhibited a growth-promoting effect on offspring goslings, as evidenced by increased body and liver weights. These findings are consistent with previous studies in pigs, rats, and broiler chickens [17,23,26]. However, the effects of betaine on cholesterol metabolism remain inconsistent across studies. Research in mammals has shown that betaine enhanced cholesterol levels in plasma, liver, and skeletal muscle [29,33,34]. Conversely, in chickens, betaine significantly reduced serum and liver cholesterol levels [16,20,21], as well as alleviated corticosterone-induced hepatic cholesterol deposition [35]. Similarly, dietary betaine supplementation has been reported to reduce hepatic cholesterol content in blunt-snout bream by enhancing bile acid metabolism [36]. However, in the goose, data on the hepatic metabolic responses of cholesterol to betaine remains scarce. In the current study, serum and hepatic TCHO concentrations were significantly decreased, while hepatic and bile TBA concentrations were significantly increased in offspring from betaine-treated goose breeders. These findings suggest that the effects of betaine on hepatic cholesterol profiles are dependent on the dietary formulations, species, and the dose of supplementation. No pathological alternations were observed in H&E-stained sections of the gosling liver tissues both at 35 d and 63 d [22], which was in line with no significant change for the biometric parameters of ALT, AST, and LDH in serum. These results were similar to previous studies in chickens [37,38]. Many studies suggest a preventive role of betaine in non-alcoholic fatty liver disease [39] and inflammation [40]. Meanwhile, betaine alleviates oxidative stress and liver damage by reducing lipid peroxidation products while enhancing antioxidant metabolites [37], and its cellular accumulation further protects against osmotic stress, maintaining normal metabolic function even under conditions of cellular inactivity [41]. Additionally, physiological and metabolic differences in different vertebrates may influence betaine absorption and utilization efficiency. As the geese in this study were raised in floor pens with males and females mixed to allow natural mating, potential paternal effects on the growth performance and metabolism of the offspring goslings cannot be excluded.

Cholesterol synthesis and transformation are critical processes for maintaining hepatic cholesterol balance. Key enzymes involved in these processes included HMGCR, SREBP2, and CYP7A1. Previous studies have indicated that betaine treatment alleviated chronic alcohol-induced hepatic cholesterol accumulation in rats by downregulating mRNA expression of *HMGCR* and *SREBP2* [42]. Similar effects were observed in offspring juvenile chickens [21]. However, in the present study, parental betaine supplementation did not affect hepatic HMGCR and SREBP2 protein expression in offspring goslings. Interestingly, a prior study showed that maternal betaine administration increased hepatic cholesterol accumulation by inhibiting CYP7A1 expression in offspring rats [29]. In contrast, our findings demonstrated that parental betaine supplementation decreased hepatic cholesterol accumulation by upregulating both mRNA and protein expression of CYP7A1, consistent with previous studies in chickens [21]. These contrasting results between mammals and birds may be explained by differences in nutrient delivery via the mother-fetus cycle, with mammals relying on the placenta and birds utilizing eggs. Collectively, the upregulation of CYP7A1 promotes the conversion of hepatic cholesterol to bile acids, ultimately reducing cholesterol content in betaine-treated offspring goslings.

Hepatic bile acid synthesis is regulated by a negative feedback mechanism in which FXR, a ligand-activated nuclear transcription factor, plays a central role. When activated by bile acids, FXR induces the expression of the small heterodimer partner (*SHP*) gene, which subsequently suppresses the transcription of liver receptor homolog-1 and CYP7A1, preventing excessive bile acid synthesis and potential toxicity in the liver [43]. In this study, parental betaine supplementation upregulated both *CYP7A1* and *FXR* gene expression in the liver of offspring goslings, while SHP gene expression remained unchanged. This suggests that betaine may enhance the hepatic bile acid synthesis independently of the FXR-SHP regulatory pathway [44,45]. Moreover, the negative feedback regulation of bile acid is generally activated only when excessive bile acids are synthesized in the liver. Given that hepatic bile acid synthesis might still within the normal physiological range in geese, therefore, the feedback suppression may not have been triggered. Furthermore, activated FXR upregulates *BSEP* gene expression, facilitating bile acid efflux from hepatocytes to bile ducts [2]. In this study, hepatic FXR protein expression was significantly elevated in the betaine-treated offspring goslings, which corresponded with increased transcription of the *BSEP* gene. Similar observations have been reported in rats [46]. These results indicate that dietary betaine addition enhances hepatic bile acid export to the biliary system, increasing bile acid concentration in the gallbladder.

Betaine serves as a substrate in one-carbon metabolism, a process crucial for maternal-fetal interactions [47], and is involved in epigenetic gene regulation via DNA methylation [28]. Previously, we found that parental betaine supplementation significantly increased hepatic global DNA 5mC methylation in the offspring goslings, together with protein expression of the methionine cycle and methyl transfer genes [22]. DNA methylation commonly occurs in gene promoter regions without altering gene sequences. In the present study, parental betaine supplementation reduced DNA methylation level on the *CYP7A1* gene promoter in the liver of offspring goslings, correlating with increasing mRNA expression. However, no alteration was detected in the methylation status of the *BSEP* gene promoter. Similarly, other studies have shown that maternal betaine supplementation programs offspring hepatic mRNA expression of related genes through DNA methylation. For instance, maternal betaine treatment caused the promoter of the hepatic *CYP7A1* gene hypomethylated, whereas that of the *SREBP2* gene was hypermethylated in offspring chickens [21]. Moreover, maternal betaine supplementation caused the transcriptional repression of the hepatic *HMGCR* gene in neonatal piglets, which was associated with CpG island hypermethylation in its promoter regions [24]. Notably, methylation status of the promoter response to methyl donors is complex and gene-specific, not all functional genes respond uniformly to methyl donor availability [32,35, 48]. In addition, gestational choline deficiency has been shown to hypomethylate the DNA methyltransferase (*DNMT1*) gene in fetal rat livers, causing global and *IGF-2* gene hypermethylation [49]. The above findings indicate that the effect of methyl donors on gene methylation is mediated through a complex and diverse regulatory mechanism.

## CONCLUSION

In conclusion, we demonstrate that parental betaine supplementation decreased hepatic cholesterol content in offspring goslings through DNA hypomethylation of the CYP7A1. Follow-up studies are necessary to investigate whether other regulatory mechanisms are involved in betaine-induced hepatic cholesterol metabolism in the goose.

## Figures and Tables

**Table 1:** Ingredients and nutrient composition of the basal diet for goose breeders and offspring goslings (as-fed basis)

	Gosling diets			

Items	Goose breeders	1 to 21 d	22 to 42 d	43 to 63 d
Ingredients (%)	100.00	100.00	100.00	100.00
Corn	48.27	36.77	37.38	43.78
Wheat	20.00	20.00	20.00	20.00
Rice bran meal	4.00	4.00	4.00	4.00
Soybean meal^1)^	14.20	28.60	25.90	16.70
Corn gluten meal	3.00	2.00	2.00	3.00
DDGS^2)^	-	3.00	4.00	5.00
Soybean oil	1.10	1.20	2.50	3.50
Limestone	7.16	1.50	1.45	1.45
Dicalcium phosphate	0.81	1.49	1.39	1.16
Methionine	0.11	0.24	0.18	0.17
Lysine	0.35	0.20	0.20	0.24
Premix^3)^	1.00	1.00	1.00	1.00
Calculated nutrient levels (%)				
Metabolizable energy (MJ/kg)	12.33	11.72	12.13	12.55
Crude protein	15.00	20.50	18.50	15.5
Crude fat	3.31	3.36	4.85	6.14
Crude ash	10.63	6.60	6.02	5.42
Calcium	3.00	1.00	0.95	0.90
Total phosphorus	0.57	0.76	0.67	0.60
Digestible lysine	0.70	1.00	0.90	0.75
Digestible total sulfur amino acids	0.55	0.73	0.66	0.56

1) The protein content of the soybean meal is 43%.

2) The protein content of DDGS is 27%.

3) Premix provided per kilogram of diet: vitamin A, 7,000 IU; vitamin D, 4,000 IU; vitamin E, 20 IU; vitamin K, 1.5 mg; vitamin B1, 2 mg; vitamin B2, 10 mg; vitamin B6, 3 mg; vitamin B12, 0.02 mg; niacin, 50 mg; pantothenic acid, 10 mg; folic acid, 1 mg; biotin, 0.2 mg; choline, 400 mg; Fe, 60 mg; Cu, 10 mg; Mn, 100 mg; Zn, 90 mg; I, 0.5 mg.

DDGS, distiller’s dried grains with soluble.

**Table 2:** Nucleotide sequences of specific primers

Target genes	Primer sequence (5’ to 3’)	Products (bp)
*PPIA* (NM_001166326.2)	F: TTACGGGGAGAAGTTTGCCG R: TGGTGATCTGCTTGCTCGTC	239
*SREBP1* (AY029224)	F: GAGACCATCTACAGCTCCGC R: CATCCGAAAAGCACCCCTCT	145
*HMGCR* (XM_013192113.1)	F: CGAGAGAGTCGTGAAGGACG R: GCTATCCACCGACTATGGGC	155
*SREBP2* (XM_040554491.1)	F: GGACAGATGCCAAGATGC R: GGTCAATGCCCTTCAACA	150
*LDLR* (NM_204452.1)	F: CCACCATTTGGCAGAGGAA R: ACCGCAGTCAGACCAGAAGAG	86
*SRB1* (XM_048073965.2)	F: TCACTTCTACAATGCTGACCCAA R: TGAGCCATCAATGTATCCACTC	241
*ABCG5* (XM_035545896.1)	F: TGCCTGACTGCAAACCAGAT R: TGAAGAGTTCTGAGCGAGGC	100
*ABCG8* (XM_013173870.1)	F: ATGTGATAACCTGCCGCGAT R: CTCGTGTTTGCACACTGACG	265
*LXRα* (HM138512.1)	F: CCCAGCCCTTCCCACAAACT R: CTGCCTCGCTTCACGGTTATTAG	159
*CYP7A1* (XM_013185866.3)	F: TGCTCCGCATGTTCCTGAAT R: AGAAGGTAAACAAGCTCCAAAAAGT	132
*CYP27A1* (XM_066999464.1)	F: ACTTTCGTCTGGCTCTTCCTG R: CATCGGGTATTTGCCCTCCT	177
*BSEP* (XM_013171440.1)	F: ATGCATCACAGGTCCAAGGG R: GACAAGGCCAAAAAGGGCAG	151
*OSTα* (XM_013195021.3)	F: GCTGGACATGGTCCAACTCA R: CACCATCATGGAACGTGGGA	208
*OSTβ* (XM_013181963.1)	F: CGAGCAGGAAAGGAGTTGGT R: TGGTAAGGGCTGCATGGAAG	138
*FXR* (XM_013182547.1)	F: ATCCTGTCCCCAGATCGACA R: TCCGTAATTCTGTGAGGCGG	157
*SHP* (XM_013199600.1)	F: TCCCTCCTGCCCTTATCACA R: TCGTACAGCATTTCCGCGAT	88
*MeDIP-PCR*		
*CYP7A1 promoter*	F: AAGTGCTGTCCTACTCCCCG R: GTAACTGCTGGAGACACACCA	125
*BSEP promoter*	F: AGGCAGAAAATGCAACATGTCTA R: GATAGCCTGGTCAGACACTCC	133
